# Single nucleotide polymorphisms of the genes *IL-2*, *IL-2RB*, and *JAK3* in patients with cutaneous leishmaniasis caused by *Leishmania* (V.) *guyanensis* in Manaus, Amazonas, Brazil

**DOI:** 10.1371/journal.pone.0220572

**Published:** 2019-08-08

**Authors:** Felipe Jules de Araújo Santos, Lener Santos da Silva, José do Espírito Santo Júnior, Tirza Gabrielle Ramos de Mesquita, Mara Lúcia Gomes de Souza, Moacir Couto de Andrade Júnior, Sinésio Talhari, Rajendranath Ramasawmy

**Affiliations:** 1 Programa de Pós-Graduação em Medicina Tropical, Universidade do Estado do Amazonas, Manaus, Amazonas, Brazil; 2 Fundação de Medicina Tropical Dr. Heitor Vieira Dourado, Manaus, Amazonas, Brazil; 3 Programa de Pós-graduação de Imunologia Básica, Universidade Federal do Amazonas, Manaus, Brazil; 4 Post-Graduation Department, Nilton Lins University, Manaus, Amazonas, Brazil; 5 Department of Food Technology, Instituto Nacional de Pesquisas da Amazônia (INPA), Manaus, Amazonas, Brazil; 6 Faculdade de Medicina, Nilton Lins University, Manaus, Amazonas, Brazil; King Saud University, SAUDI ARABIA

## Abstract

Leishmaniasis is a disease caused by intracellular protozoan parasites of the genus *Leishmania*. In endemic areas, only a portion of exposed subjects develops cutaneous leishmaniasis (CL), suggesting that the genetic inheritance of the host plays a vital role in both resistance and susceptibility to the disease. Interleukin-2 (IL-2) is a cytokine that plays a central role in the regulation of the immune response in infection through the axis IL-2/IL-2R (receptor) complex, triggering a series of intracellular events, among which the signaling of Janus kinase/signal transducers and activators of transcription (JAK-STAT). The present study aimed at verifying the possible relationship between single nucleotide polymorphism (s) (SNP s) in the genes *IL-2*, *IL-2RB*, and *JAK3* in subjects with CL caused by *Leishmania guyanensis* in the city of Manaus, state of Amazonas, Brazil. 820 patients with CL and 850 healthy subjects (control group) coming from the same endemic areas as the patients were examined. The SNPs -2425G/A (rs4833248) and -330 T/G (rs2069762), located in the *IL-2* gene promoter region, seem to influence the expression of the gene and the SNP +10558G/A (rs1003694) and +13295T/C (rs3212760) located in the 3rd intron of the *IL-2RB* gene and the 13th intron of the *JAK3* gene, respectively, were studied by PCR-RFLP. Genotypes and alleles frequencies were obtained by direct counting. For the comparison between the two groups, the χ2 test with OR (odds ratio) and the 95% confidence interval (CI) were used. Similar genotypes and alleles frequencies for the different SNPs were observed in both patients with CL and healthy controls. Comparison of genotypic and allelic frequency between patients with CL and healthy subjects did not show any difference. These polymorphisms do not predict susceptibility to, or protection against the development of CL caused by *L*. *guyanensis* in the Amazonas.

## Introduction

Leishmaniasis is a vector-borne disease caused by intracellular protozoa of the genus *Leishmania* [1]. Infections caused by these parasites have a wide clinical spectrum, including cutaneous leishmaniasis (CL), mucosal leishmaniasis (ML), and visceral leishmaniasis (VL) [2]. These diseases are endemic in 98 tropic and subtropic countries with an estimated incidence of 0.2–0.4 million of VL and 0.7–1.2 million of CL each year [3]. In the city of Manaus, capital of the state of Amazonas, Brazil, the cutaneous form of leishmaniasis is mainly caused by *L*. *guyanensis* [4]. Of note, the major species that cause American Tegumentary Leishmaniasis in Brazil are *L*. *braziliensis*, *L*. *guyanensis*, *L*. *lainsoni*, *L*. *amaozonensis*, *L*. *shawi*, *L*. *naiffi* and *L*. *lindbergi*.

Components of innate and adaptive immunity, such as phagocytic cells, natural killer (NK), CD4^+^, CD8^+^ lymphocytes and regulatory T cells (Treg), are associated with the control of leishmaniasis [5,6]. Immunocompetent cells secrete inflammatory cytokines (tumor necrosis factor alpha (TNF-α), interferon gamma (IFN-γ), and Interleukin -17) and regulatory cytokines (IL-10, IL-27, and transforming growth factor beta 1 (TGF-β1) that are involved in the modulation of the immune response in patients with CL and ML [7]. These studies argue that the balance between the components of the immune system may be crucial for the killing of *Leishmania* without causing tissue damage in the vertebrate host.

Interleukin-2 (IL-2) is a cytokine that plays a central role in the regulation of the immune response through the axis IL-2/IL-2R (receptor) complex, triggering a series of intracellular events, among which the signaling of Janus kinase/signal transducers and activators of transcription (JAK-STAT) [8]. The IL-2R is composed of three subunits: IL-2Rα (CD25), IL-2Rβ (CD122), and γ_c_ (common) (CD132) [9]. Dysregulated IL-2 signaling results in the death of Treg cells leading to the rapid development of various autoimmune diseases. Knockout mice for CD25 or CD122 develop lethal autoimmune diseases because of lack of Treg cells [10, 11].

Conversely in Leishmaniasis, CD4^+^CD25^+^ Treg cells are reported to suppress the ability of CD4^+^CD25^-^ effector T cells to eliminate the parasites *L*. *major* at the site of infection in C57BL/6 mice [12]. Furthermore, neutralization of IL-2 but not interferon gamma in BALB/c mice infected with *L*. *donovani* increased the parasite load [13]. In experimental VL, BALB/c mice infected with *L*. *donovani* and treated continuously with administered IL-2 reduced the parasite burden compared to control mice through the induction of interferon gamma [14].

Peripheral blood mononuclear cells (PBMCs) from patients with CL caused by *L*. *braziliensis* stimulated with Leishmania antigen and treated with neutralizing antibodies to IL-2 lead to decrease interferon gamma production [15] that is important for macrophage activation and clearing of the parasite. Intra-nodular injection of recombinant IL-2 in patients with disseminated CL (DCL) reduced parasite numbers, associated with CD4+T cell infiltration [16]. Several transcripts of IL-2 pathway are also observed in cutaneous lesions of patients caused by *L*. *braziliensis* [17]. Recently, IL-2 was identified as a major upstream signaling molecule in CD4+ T cells from VL patients with active disease and selectively promote antigen-specific IFN-γ production by blood cells from VL patients [18].

Susceptibility to VL caused by *L*. *donovani* in Sudan is linked to chromosome 22, the 22q12 locus [19], and in this locus, polymorphisms in the gene encoding the IL-2Rβ chain are associated with the development of VL [20]. Additionally, several polymorphisms of the genes *IL-2*, *IL-2RA*, *IL-2RB*, and *JAK3* were related to CL [17].

According to Castellucci et al. [21], many genes contribute to the risk of developing CL. Given the predominant role played by IL-2, we hypothesized that polymorphisms present in genes involved in the IL-2/IL-2R pathway signaling could influence the host susceptibility to the development of CL caused by *L*. *guyanensis* in the city of Manaus, state of Amazonas, Brazil.

The following tag-SNPs rs4833248, rs2069762, rs1003694 and rs3212760 were chosen from the HAPMAP according to their minor allele frequencies (MAF) ≥ 15% in different populations such as Europeans, Mexican, Peruvian and Africans to avoid spurious associations with our sample size. The frequencies from these populations were chosen, as our study population is an admixture of nearly 50 to 60% of Native American, 40 to 50% European and around 10% African ancestry [22]. The SNPs -2425G/A (rs4833248) and -330 T/G (rs2069762) are located in the *IL-2* gene promoter region and the SNP rs2069762 is suggested to influence the expression of the gene [23,24]. The SNP +10558G/A (rs1003694) and +13295T/C (rs3212760) are located in the 3rd intron of the *IL-2RB* gene and the 13th intron of the *JAK3* gene, respectively.

## Materials and methods

### Area of study and population

This study was carried out in regions of the city of Manaus, capital of the state of Amazonas, Brazil, specifically in the areas surrounding BR-174 and AM-010 that became endemic for the infection by *L*. *guyanensis* (i.e., communities of Pau-Rosa, Cooperativa, Água-Branca, Leão, and Brasileirinho). The state of Amazonas is situated in the extreme north of Brazil at latitude -19,9657 and longitude -44,0413. Patients with active CL (less than six lesions) infected with *L*. *guyanensis* were recruited at the Fundação de Medicina Tropical Dr. Heitor Vieira Dourado (FMT-HVD) from November 2012 to April 2017, a regional reference center for treatment of leishmaniasis. The control group of the study comprises healthy subjects from the same endemic area as the patients, thereby sharing similar eco-epidemiology for more than ten years. The healthy subjects have no history of leishmaniasis, and most of them are agriculture workers as the patients. Most of the individuals participating in the study declared a history of malaria as these regions also are endemic areas of vivax malaria. This study population is an admixture of native American ancestry and is called Caboclo.

### Ethical approval

This study was carried out in accordance with the Helsinki Declaration and approved by the Research Ethics Committee of the FMT-HVD/2012 (CAAE—Certificado de Apresentação para Apreciação Ética: 09995212.0.0000.0005). All volunteers provided written informed consent for sample collection and subsequent analysis. For patients younger than 18 years of age, the parents or responsible party signed the Informed Consent Form, consenting to the child’s participation. This study was performed in accordance with the guidelines strengthening the reporting of genetic association studies (STREGA) [25].

### Identification of *Leishmania* sp. and DNA extraction

Parasite DNA was extracted from biopsies specimens of the lesions of patients. The discrimination of the *Leishmania viannia* subgenus specific PCR was in accordance with established protocols [26, 27]. All volunteers who participated in this project were submitted to venipuncture to collect 5 mL of peripheral blood in vacutainer containing ethylenediaminetetraacetatic acid–EDTA, (Becton Dickinson). Genomic DNA was extracted by the salting out method [28].

### Molecular analysis of polymorphisms

The different SNPs (rs4833248) and rs2069762 of *IL-2*, rs1003694 of *IL-2RB* and rs3212760 of *JAK3*) were identified by PCR-RFLP. The respective restriction enzymes and the alleles size fragments are shown in Table 1. The PCR cycles and the primers for each SNP as well as the conditions for amplification of the DNA fragments are also shown in Table 1. PCR reactions were performed in the Eppendorf Mastercycler ep, and consisted of 50 ng of genomic DNA in a final volume of 25 μL containing 1.5 mmol/L of MgCl_2_, 0.25 pmol/L of forward and reverse primers, 40 μmol/L of each dNTP (dideoxynucleotide triphosphate) and 2 U of *Taq polymerase* in buffer containing 100 mmol/L of Tris-HCL (pH 8.3) and 500 mmol/L KCL. 10 μL of PCR product was digested with 5U of restriction enzyme for three hours (New England Biolabs) in a final volume of 20 μL containing 2 μL of 10X enzyme buffer, according to the manufacturer's instructions and submitted in a 3% agarose gel electrophoresis stained with ethidium bromide. Allelic discriminations were according to the size of fragments resulting from the restriction digestion.

**Table 1:** Primers and PCR conditions for the studied polymorphisms.

Primer sequence, 5’-3’ ^a^	Restriction enzyme	PCR^b^ protocol	Allele; length in bp^d^
***IL2—*rs4833248** **F^c^: 5’- GGCCTCTTGGGTTTTCAAGATTCAG-3’** **R^c^: 5’-TGGTGTTTCATTATGGAGGGCC-3’**	*Dde I*	95°C for 5 min, 40 x (95°C for 30 s, 59°C for 30 s, 72°C for 30 s), 72°C for 7 min	Allele A 136 +53 Allele G 189
***IL2—*rs2069762** **F^c^: 5’-CAGGAAACCAATACACTTCCTGTT-3’** **R^c^: 5’-GTAACTCAGAAAATTTTCTTGGTC-3’**	*Ava II*	95°C for 5 min, 40 x (95°C for 30 s, 56°C for 30 s, 72°C for 30 s), 72°C for 7 min	Allele G 117 + 24 Allele A 141
***IL2RB*—rs1003694** **F^c^: 5’- GCTTGGATTTTGAGAGACCCT-3’** **R^c^: 5’-CCACCTCTCTGTGGTCTTCCTCTT-3’**	*RsaI*	95°C for 5 min, 40 x (95°C for 30 s, 56°C for 30 s, 72°C for 30 s), 72°C for 7 min	Alelle A 121+63 Alelle G 184
***JAK3*—rs3212760** **F^c^: 5’- GGAGGGCAGCACGGAGCATTGTGGA-3’** **R^c^: 5’-GTCCTCTGCCTAGAACACCCCTC-3’**	*HpyCH4III*	95°C for 5 min, 40 x (95°C for 30 s, 66,9°C for 30 s, 72°C for 30 s), 72°C for 7 min	Allele C 129 + 27 Allele T 156

^a^ 5’-3’: nucleic acids in the 5'-3' direction

^b^ PCR: Polymerase Chain reaction

^c^ F and R: Primers forward and reverse

^d^ bp: base pairs

### Statistical analysis

The site http://ihg.gsf.de/cgibin/hw/hwa1 was used for logistic regression genetic analysis For comparison of cases with the control groups, the two-tailed χ^2^ test was used along with OR and 95% confidence interval. The Hardy-Weinberg equilibrium (HWE) was determined by comparing the observed and expected genotype frequencies. Linkage disequilibrium was performed using Haploview 4.2 software, estimating degree of linkage disequilibrium (LD) between alleles.

## Results

### Study population

The study included 820 patients with CL caused by *L*. *guyanensis* and 850 healthy subjects without a history of CL. Of the 820 patients with CL, 609 (74%) were males with a mean age of 34.6 ± SD 13.7 years and 211 (26%) females (mean age of 37.4 ± SD 15.6 years). Among the healthy subjects, 578 (68%) were males with a mean age of 42 ± SD 17.5 years and 272 (32%) females (mean age of 39.6 ± SD 17.1). The distribution of sexes was significantly different between the two studied groups (p <0.05).

### Frequency of genotypes and alleles

The frequency distribution of the genotypes and alleles for the studied SNPs are presented in Table 2. There was no evidence of HWE deviation for all the SNPs in both groups. Concerning the results of the two polymorphisms in the promoter region of the *IL-2* gene, the frequency of genotypes GG, GA, and AA of the rs4833248 SNP were 52%, 39%, and 9%, respectively, among the patients and in the healthy group, 54%, 37%, and 9%, respectively (p = 0.3). The SNP rs2069762 showed that the TT, TG, and GG genotypes had 50%, 43%, and 7%, respectively, and in the healthy group, 53%, 39%, and 8%, respectively (p = 0.5). The rs1003694 SNP in the *IL-2RB* gene showed that the GG, GA, and AA genotypes had 52%, 39%, and 9%, respectively, among the patients. In the controls, they were 49%, 41%, and 10%, respectively (p = 0.4). Then, the rs3212760 SNP in the *JAK3* gene had the frequency of the TT, TC, and CC genotypes of 53%, 40%, and 7%, respectively, among the patients. In the healthy group, 56%, 36%, and 8%, respectively (p = 0.6). Comparison of the different genotypes and alleles of the SNPs studied between patients with CL and healthy group revealed no statistical difference as shown in Table 2.

**Table 2:** Genotype and allele frequencies for the polymorphisms in patients with CL and healthy controls.

Genotype		Cases (%)	Healthy group (%)	comparisons	p-value	OR^a^ [95%CI]^b^
		**n = 748**	**n = 759**			
*IL2—*rs4833248	G/G	386 (52)	416 (54)	GG vs. AA	0.5	1.1 [0.7–1.6]
	G/A	295 (39)	278 (37)	GG vs. GA	0.2	1.1 [0.9–1.4]
	A/A	67 (9)	65 (9)	GG vs. GA + AA	0.2	1.1[0.9–1.4]
	G	1.067 (71)	1.110 (73)	G vs. A	0.2	1.0 [0.9–1.3]
	A	429 (29)	408 (27)			
		**n = 734**	**n = 762**			
*IL2*—rs2069762	T/T	362 (50)	400 (53)	TT vs. GG	0.6	0.9 [0.6–1.3]
	T/G	319 (43)	298 (39)	TT vs. TG	0.1	1.2 [0.9–1.5]
	G/G	53 (7)	64 (8)	TT vs. TG + GG	0.2	1.1 [0.9–1.4]
	A	1.043 (71)	1.098 (72)	T vs. G	0.5	1.1 [0.9–1.2]
	G	425 (29)	426 (28)			
		**n = 700**	**n = 715**			
*IL2RB*—rs1003694	G/G	360 (52)	350 (49)	AA vs. GG	0.5	1.0 [0.6–1.2]
	G/A	274 (39)	293 (41)	AA vs. GA	0.4	1.0 [0.7–1.1]
	A/A	66 (9)	72 (10)	AA vs. GA + GG	0.3	1.0 [0.7–1.1]
	G	994 (71)	993 (69)	A vs. G	0.4	1.0 [0.7–1.1]
	A	406 (29)	437 (31)			
		**n = 616**	**n = 735**			
*JAK3—*rs3212760	T/T	324 (53)	408 (56)	TT vs. CC	0.7	1.0 [0.6–1.4]
	T/C	247 (40)	266 (36)	TT vs. TC	0.2	1.2 [0.9–1.5]
	C/C	45 (7)	61 (8)	TT vs. TC + CC	0.3	1.1 [0.9–1.4]
	T	895 (73)	1.082 (74)	T vs. C	0.5	1.0 [0.9–1.2]
	C	337 (27)	388 (26)			

^a^ OR: odds ratio

^b^ 95% CI: 95% confidence interval

As there was an excess of females in the control group, we stratified the groups according to sex and repeat the comparison. None of the SNPs shows an association with the development of CL (S1 Table) and similar frequencies of genotypes and alleles between and females were observed suggesting that sex does not have an influence on either susceptibility or protection to *L*. *guyanensis*-infection. Furthermore, all the SNPs were in HWE in both males and females.

### Analysis of haplotypes

LD analysis showed a moderate correlation between the two SNPs in the *IL-2* gene promoter region (rs4833248 and rs2069762), D’ of 0.7 with confidence limits of 0.68 to 0.74, while the *r*^*2*^ values is 0.5. Four haplotypes GT, AG, GG, and AT were observed and no significant differences were found between the two groups (Table 3).

**Table 3:** Haplotypes of the promoter region of *IL-2* present in the study population.

Haplotype	*IL-2*–2811 G/A rs4833248	IL-2–330 T/G rs2069762	Case (%)	Control (%)	*p*-value
**1**	G	T	1064 (65)	1131 (67)	0.4
**2**	A	G	367 (23)	371 (22)	0.7
**3**	G	G	101 (6)	106 (6)	0.9
**4**	A	T	100 (6)	92(5)	0.4

## Discussion

In leishmaniasis, the genetic background of the host influences the balance between pro- and anti-inflammatory responses mediated mainly by cytokines released by immune cells [29,30]. In animal model, outcome of *Leishmania* infection is influenced by IL-2 production. Recombinant IL-2 (rIL-2) induces antileishmanial activity through the production of IFN-γ in *L*. *donovani* infected BALB/c mouse [15]. In *L*. *donovani* infection, IFN-γ performs protective functions in contrast to IL-2, which induces immunoregulatory cells mediated by IL-10 [31]. In the first week of *L*. *major* infection in murine models, CD4^+^T cells produced a wide range of cytokines, including IL-2 and IFN-γ [32]. These cytokines performed protective immunity during this initial phase of the infection. On the other hand, IL-2 blockade in BALB/c mice infected with *L*. *major* enhanced protection via the differentiation of T helper (Th1) [33].

In endemic areas, whole blood stimulation from *L*. *infantum* asymptomatic subjects with soluble leishmanial antigen showed significant increase of IL-2 [34]. PBMC from treated patients with VL, the levels of IL-2 and IFN-γ stimulated by soluble *Leishmania* antigen (SLA) can be used in the monitoring of cellular immunity as cure markers [35]. PBMCs from healthy individuals, when stimulated with antigens from *L*. *guyanensis*, showed that Treg cells produced TGF-β1, which in turn inhibited IL-2 [36]. CL and ML are characterized by a predominance of Th1 (IFN-γ, TNF-α, and IL-2) with intense inflammatory response in lesions with few parasites [14]. Altogether, these studies have shown the importance of IL-2 in *Leishmania* infection.

Considering the strong inflammatory action in leishmaniasis, the present study was designed to evaluate the association between SNPs of the IL-2 pathway and the susceptibility to the development of CL caused by *L*. *guyanensis*. However, no association was found between these polymorphisms (rs4833248, rs2069762, rs1003694 and rs3212760) and the development of CL, that is, no differences of genotypes and alleles frequencies were observed between CL cases and the control groups.

Despite the absence of association with CL, the large sample size (820 cases and 850 controls) of the present study provides confidence that these studied SNPs can be discarded in susceptibility studies of *L*. *guyanensis*-infection. These data were similar in patients with CL infected with *L*. *braziliensis* from Northeast Brazil (Bahia), according to their minor allelic frequencies of *IL-2* rs4833248/A (0.21) and rs2069762/G (0.21), *IL-2RB* rs1003694/A (0.32), and *JAK3* rs3212760/C (0.34) and no association with the development of CL was observed [17]. Our data are in agreement with the latter study. Notably, the population of Bahia is mainly an admixture of European (36.3%), African (49.2%) and Amerindian (14,5%) ancestry [37] compared to our study population which is of origin of nearly 50 to 60% of Native American, 40 to 50% European and around 10% African ancestry [22]. The causing agent of CL is mainly *L*. *braziliensis* in Northeast Brazil and *L*. *guyanensis* in the Amazonas. The replication of this study with a different population and etiological agent showed that these SNPs have no influenced on the development of CL irrespective of the *Leishmania* species and population ethnicity.

In the present study, allele frequencies displayed a certain similarity with the study design of 1000 Genomes Project [38]. The MAF of allele A to rs4833248 observed in this population was 0.27, similar to the Japanese (0.27), Utah European (0.26), Mexican (0.34), Peruvian (0.35) and the European populations (0.29), but significantly different to the African population (0.03). The MAF of the G allele for rs2069762 was 0.28, similar to the European (0.29), Utah European (0.26), Japanese (0.27), Mexican (0.34), Peruvian (0.35) and the East Asian population (0.32), but differed to the African population (0.03). The allele A of rs1003694 was 0.31, similar to the Mexican (0.33), Utah European (0.27), South Asia (0.29), and European (0.27) populations. The C allele of rs3212760 had a frequency of 0.26, similar to the Indian (0.28), Mexican (0.31), Italian (0.30) and African (0.33) populations.

Previous *in vitro* studies have suggested that the -330T/G rs2060762 G/G genotype was associated with high levels of IL-2 [23]. In Iranian subjects, the T/T genotype was responsible for the low levels of IL-2, while the G/T and G/G genotypes were related to normal cytokine levels [24].

SNP rs1003694 in the *IL-2RB* gene showed no association with CL. However, other polymorphisms located in the proximal domain of the cytoplasmic receptor base alter the phosphorylation site in which JAK1 and JAK3 bind [20] and are worse of investigation. *Leishmania* parasites may block JAK/STAT signaling, compromising the production of nitric oxide (NO) [39], important for elimination of parasites. Furthermore, the amastigote form of *L*. *donovani* blocks the signaling of JAK and STAT1 and thus decrease IFN-γ levels [40]. *L*. *donovani* promastigote releases glycoprotein 63 (gp63) into the cytoplasm and rapidly induces the activation of a tyrosine phosphatase, which subsequently inhibits the phosphorylation of JAK [41]. It is reported that the down-regulation of toll-like receptors (TLR) and JAK/STAT genes in NK cells is associated with CL caused by *L*. *mexicana* [42]. These reports reveal the importance of the IL-2 signaling pathway in driving the inflammatory process in *Leishmania-*infection.

## Conclusions

Two SNPs located on the *IL-2RA* gene were cited to be independently associated with CL susceptibility [17]. Therefore, polymorphisms in other components of the IL-2 pathway may be related to *L*. *guyanensis* infection. Altogether, the present study showed no association of target SNPs (rs4833248, rs2069762, rs1003694, and rs3212760) in the IL-2/IL-2R axis and susceptibility to *L*. *guyanensis* infection. It is likely that the genetic effect of the host with CL is due to multiple genes. Therefore, it will be interesting to perform more studies of other polymorphisms present in the IL-2/IL2R axis.

## Supporting information

S1 Table Genotype and Allele frequencies of the rs4833248, rs2069762, rs1003694, and rs3212760 in the study population stratified by sex. (DOCX)
