# Supplementary material for: Single nucleotide polymorphisms of the genes IL-2, IL-2RB, and JAK3 in patients with cutaneous leishmaniasis caused by Leishmania (V.) guyanensis in Manaus, Amazonas, Brazil
Source: PLoS One. 2019 Aug 8;14(8):e0220572. doi: 10.1371/journal.pone.0220572 (PMC6687158; doi:10.1371/journal.pone.0220572)
Supplement: S1 Table — (DOCX) [file pone.0220572.s001.docx]

**S1** **Table.** Genotype and Allele frequencies of the rs4833248, rs2069762, rs1003694, and

rs3212760 in the study population stratified by sex.

|  | **Patients with CL, no. (%)** | | **Controls, no. (%)** | | |
| --- | --- | --- | --- | --- | --- |
|  | **Males** | **Females** | **Males** | **Females** |  |
| **rs4833248** | n= 557 | n= 191 | n= 519 | n= 240 |  |
| **Genotypes** |  |  |  |  |  |
| **GG** | 287 (52) | 99 (52) | 291 (56) | 125 (52) |  |
| **GA** | 217 (39) | 78 (41) | 182 (35) | 96 (40) |  |
| **AA** | 53 (9) | 14 (7) | 46 (9) | 19 (8) |  |
| **Alelle** |  |  |  |  |  |
| **G** | 791 (71) | 276 (72) | 764 (74) | 346 (72) |  |
| **A** | 323 (29) | 106 (28) | 274 (26) | 134 (28) |  |
| **rs2069762** | n= 540 | n= 194 | n= 512 | n= 250 | |
| **Genotypes** |  |  |  |  | |
| **TT** | 267 (50) | 95 (49) | 263 (51) | 137 (55) | |
| **TG** | 229 (42) | 90 (46) | 204 (40) | 94 (38) | |
| **GG** | 44 (8) | 9 (5) | 45 (9) | 19 (7) | |
| **Alelle** |  |  |  |  | |
| **T** | 763 (71) | 280 (72) | 730 (71) | 368 (74) | |
| **G** | 317 (29) | 108 (28) | 294 (29) | 132 (26) | |
| **rs1003694** | n= 517 | n= 183 | n= 486 | n= 229 | |
| **Genotypes** |  |  |  |  | |
| **GG** | 277 (54) | 83 (45) | 230 (47) | 120 (52) | |
| **GA** | 192 (37) | 82 (45) | 211 (44) | 82 (36) | |
| **AA** | 48 (9) | 18 (10) | 45 (9) | 27 (12) | |
| **Alelle** |  |  |  |  | |
| **G** | 746 (72) | 248 (68) | 611 (67) | 322 (70) | |
| **A** | 288 (28) | 118 (32) | 301 (33) | 136 (30) | |
| **rs3212760** | n= 458 | n= 158 | n= 493 | n= 242 | |
| **Genotypes** |  |  |  |  | |
| **TT** | 248 (54) | 76 (48) | 268 (54) | 140 (58) | |
| **TC** | 181 (40) | 66 (42) | 181 (37) | 85 (35) | |
| **CC** | 29 (6) | 16 (10) | 44 (9) | 17 (7) | |
| **Alelle** |  |  |  |  | |
| **T** | 677 (74) | 218 (69) | 717 (73) | 365 (75) | |
| **C** | 239 (26) | 98 (31) | 269 (27) | 119 (25) | |
